# The trends in the use of psychopharmacological medications in Ukraine 2010–2022

**DOI:** 10.1186/s12888-026-07835-2

**Published:** 2026-01-23

**Authors:** Valeriia Zarubina, Lone Holst, Ingunn Marie Stadskleiv Engebretsen

**Affiliations:** 1 Centre for International Health, Department of Global Public Health and Primary Care, University of Bergen, Bergen, Norway; 2 Research Group in Social Pharmacy, Department of Global Public Health and Primary Care, University of Bergen, Bergen, Norway

**Keywords:** Psychopharmacology, Antidepressants, Anxiolytics, Hypnotics, Psychostimulants, Antipsychotics, Mental health, Ukraine

## Abstract

**Background:**

Between 2010 and 2022, Ukraine experienced multiple societal disruptions, including healthcare reforms, the COVID-19 pandemic, and the full-scale war in 2022. These events may have influenced mental health needs and access to psychopharmacological treatment across the country.

**Objective:**

This study aimed to describe trends in the use of outpatient psychopharmacological medicines in Ukraine from 2010 to 2022, as measured by the annual number of dispensed packages from community pharmacies.

**Methods:**

We conducted a register-based, retrospective analysis of community pharmacy sales data from the PharmXplorer database^®^, maintained by Proxima Research LLC. Annual numbers of packages dispensed for antidepressants, anxiolytics, hypnotics and sedatives, psychostimulants and antipsychotics (ATC N05A-C, N06A-B) were analysed using joinpoint regression on log-transformed counts. Prescription volumes by sex and age were described for 2015–2022. Statistical analyses were performed using Stata SE 18 and the Joinpoint Regression Program version 5.4.0.

**Results:**

Overall psychopharmacological use declined between 2010 and 2022, with drops in 2020 and 2022. Joinpoint regression showed a sustained decline in hypnotics and sedatives (N05C; average annual percent change [AAPC] -4.9% [95% CI -5.5% to -4.2%]; *p* < 0.001). Psychostimulants (N06B) showed short-term fluctuations with no significant overall long-term trend (AAPC − 0.7% [95% CI -2.3% to 0.5%]; *p* = 0.21). Anxiolytics (N05B) displayed a modest yet statistically significant long-term increase (AAPC 2.6% [95% CI 0.7% to 4.4%]; *p* = 0.006). Antipsychotic use (N05A) also increased (AAPC 3.9% [95% CI 2.5% to 5.3%]; *p* < 0.001), with a steeper rise from 2016. Antidepressant use (N06A) increased overall (AAPC 7.2% [95% CI 5.0% to 9.8%]; *p* < 0.001), driven by a marked rise after 2015. By the end of 2022, escitalopram and gidazepam were the most widely dispensed antidepressant and anxiolytic, respectively.

**Conclusions:**

This study describes long-term trends in outpatient psychopharmacological medicine use in Ukraine from 2010 to 2022. We observed changes in prescription volumes over 2015–2022, shifts in the distribution of pharmacy-dispensed medicines across ATC classes during 2010–2022, and declines in overall utilisation during major disruptions to health service delivery. These findings can support monitoring of psychopharmacological medicine utilisation and mental health service planning.

**Clinical trial registration number:**

Not applicable.

**Supplementary Information:**

The online version contains supplementary material available at 10.1186/s12888-026-07835-2.

## Background

Mental and behavioral disorders are a major contributor to the global burden of disease, with their prevalence steadily rising over the past decades. According to the World Health Organization (WHO), mental disorders accounted for approximately 13% of the global disease burden in 2011, with higher proportions in low- and middle-income countries when measured in disability-adjusted life years (DALYs) [1]. Depression is projected to become the leading cause of disease burden worldwide by 2030 [2]. More recent estimates from the WHO suggest that one in eight people globally is affected by a mental disorder, yet most countries allocate less than 2% of their health budgets to mental healthcare [3].

The 2019 Global Burden of Disease data highlight anxiety disorders (ICD F40–F41) and depressive disorders (ICD F32) as the most prevalent mental conditions globally [3]. This trend is mirrored in Ukraine, where the mental health system has faced longstanding structural challenges, including insufficient access to care, inadequate early detection, and limited integration of mental health services into primary care [4]. In 2017, the Ukrainian government adopted the “Concept for the Development of Mental Health Care for the Period until 2030” based on WHO recommendations, aiming to strengthen mental health services and integrate them into community-based platforms [5].

According to the World Bank’s 2017 evaluation, nearly one-third of Ukrainians have experienced mental disorders in their lifetime. Gender-based differences were observed: depressive and anxiety disorders were more common in women, while men more frequently experienced alcohol use disorder and post-traumatic stress disorder (PTSD). The prevalence of depressive disorders in Ukraine was 6.3% (7.4% in women vs. 5.0% in men), and anxiety disorders affected 3.2% of the population (3.8% in women vs. 2.5% in men) [4].

Since 2017, Ukraine has also undergone broader health system reforms, including the establishment of the National Health Service of Ukraine, the launch of the national eHealth system, and the introduction of medicines reimbursement through the “Affordable Medicines Programme” [6–8]. These reforms were designed to improve transparency and efficiency in health financing and to expand access to essential medicines, including for chronic non-communicable diseases. However, despite these developments, Ukraine still does not have a single comprehensive national register that captures all outpatient medicine usage across the country. Routine information systems and newly launched dashboards currently provide detailed data, mainly for reimbursed medicines and e-prescriptions, while large segments of the retail market remain outside these systems [9]. As a result, analyses of psychopharmacological medicine use at the national level must rely on commercial sales such as the PharmXplorer database^®^, which integrates prescription volumes (RxTest) and pharmacy sales (Sell-Out) across major urban areas.

Ukraine’s mental health landscape has also been deeply influenced by prolonged armed conflict. Research indicates that approximately 22.1% of conflict-affected populations suffer from mental disorders, including PTSD, depression, and psychosis [10]. War-related factors such as population displacement, trauma exposure, and socioeconomic instability increase the risk of mental illness. Similar findings were observed in conflict zones such as Kosovo, Afghanistan, and Syria, where war conditions led to a significant rise in PTSD and depression among civilians [11–14]. In the case of Syria, 44% of young adults displayed signs of a probable severe mental disorder, and 36.9% exhibited all PTSD symptoms [15].

Given these structural, geopolitical, and public health challenges, understanding trends in the use of psychopharmacological medications in Ukraine is critical. Such analysis offers insight into how healthcare systems respond to shifting mental health demands, particularly during crises such as the COVID-19 pandemic and the full-scale war that began in 2022.

This study aims to address this gap by examining longitudinal trends in the use of psychopharmacological medications in Ukraine from 2010 to 2022. It focuses on key drug classes classified under the Anatomical Therapeutic Chemical (ATC) system, including antipsychotics (N05A), anxiolytics (N05B), hypnotics/sedatives (N05C), antidepressants (N06A), and psychostimulants (N06B), and analyzes how usage patterns changed in the context of systemic health reforms and national crises.

## Methods

### Study design

This was a register-based study with an annual assessment of psychopharmacological medication sales for the 13 years from 2010 to 2022 in Ukraine. Data were obtained from a private pharmacy register and the information-analytical database known as PharmXplorer, owned by Proxima Research LLC (2009–2023). Throughout this paper, the term ‘PharmXplorer database^®^’ refers to this proprietary platform.

The PharmXplorer database^®^ is a sales register that integrates data on pharmaceutical sales, diagnoses coded according to the International Classification of Diseases 10th Revision (ICD-10), and prescriptions in Ukraine. The database is supported by a wide range of pharmaceutical companies with representative offices in Ukraine, including those marketing products on the Ukrainian pharmaceutical market.

The PharmXplorer^®^ provides geographical coverage for prescription data monitoring and pharmacy sales across 28 cities in government-controlled (unoccupied) territories of Ukraine. The dataset reflects changes in cities affected by war or occupation. For example, severely affected cities such as Mariupol or Kherson were replaced in 2022 with referent cities (Mukachevo, Uman, Kamenetz-Podolsk, Bila Tserkva, and Kamianske). These referent cities were selected because they maintain a stable population and a comparable number of doctors, ensuring data consistency over time. The database covers only urban areas and does not include data from rural regions. Given that Ukraine has 461 cities in total, this study focuses on a subset of major urban centers where pharmaceutical data collection is feasible.

### Data collection

The PharmXplorer database^®^ integrates data from two key projects: RxTest and Sell-Out. RxTest is a syndicated project that monitors prescription data. Sell-Out tracks retail sales from community pharmacies and captures the annual number of packages dispensed to patients in the outpatient sector. These data reflect medicines that have actually left the pharmacy (i.e. prescriptions filled for prescription-only medicines and purchases of over-the-counter products), rather than wholesale distribution or hospital stock.

For the main analyses of utilisation trends, we extracted annual counts of psychopharmacological medication packages dispensed from community pharmacies from January 2010 to December 2022, based on Sell-Out data. These data reflect the volume of medicine sold in pharmacies across major urban areas and provide insights into real-world outpatient use.

To provide additional context on prescription patterns and patient characteristics, we also used data from the RxTest project. RxTest covers 16 outpatient and hospital specialties, including allergology/pulmonology, neurology, psychiatry, anesthesiology, otolaryngology, general practice/family medicine, gastroenterology, orthopedics/traumatology, urology, obstetrics/gynecology, ophthalmology, surgery, dermatology/venereology, paediatrics, endocrinology, and cardiology. RxTest data were available only for the period 2015–2022; consequently, analyses based on RxTest are restricted to these years. This broad speciality coverage ensures that the data reflect a wide spectrum of medical practices. In this study, RxTest data were used only to describe psychopharmacological prescription volumes and the distribution of patients by sex and age, whereas all trend analyses of utilisation were based on pharmacy sales (dispensed packages) from the Sell-Out data.

Information on patient profiles, diagnoses, and prescriptions of psychopharmacological medications is aggregated into the register through surveys. The survey methodology involves conducting telephone interviews with doctors or filling out online questionnaires. During the survey, respondents are asked open-ended questions, i.e., questions without any prompts or predetermined options. For instance, the survey asks healthcare professionals to describe the type of patients with chronic conditions they most commonly see in their office and to list the medicines they prescribed during the last week, including the brand name of each medicine. In RxTest, doctors report medicines they prescribed, and these data reflect prescriptions issued rather than medicines actually collected by patients. Surveys are conducted monthly, yielding a quarterly sample of 3,870 respondent doctors in 2022 and an annual total of 15,480 doctors. The survey data are extrapolated to the general population of healthcare professionals in 25 cities and, since July 2022, in 28 cities.

In 2022, the number of doctors increased from 3,766 to 3,870, primarily due to the effects of war and city replacements, which also led to an increase in the quarterly patient sample from 74,105 to 76,150. The structure of doctors in terms of their specialties and numbers has changed since 2014, largely influenced by the movement of doctors both inside and outside of the organization. Each doctor has their own identifier called AXIOMA ID, which is used to store all their information. The PharmXplorer database^®^ is updated daily to capture resignations, deaths, changes in workplace, and changes in main or additional specialties, as well as the addition of new doctors.

### Ethics

This study used anonymized, aggregated pharmacy sales data and did not involve identifiable individual-level health information. Data handling complied with applicable Ukrainian regulations. A review of ethics approval requirements was submitted to the Regional Committees for Medical and Health Research Ethics (REK) in Norway (Ref. 629798). In the decision dated 27 June 2023, REK stated that the project falls outside the scope of the Health Research Act (Sects. 2 and 4); consequently, the application was not processed, and ethical approval was not required. The PharmXplorer database^®^ (Proxima Research LLC) does not contain personally identifiable information accessible to the researchers. Data access was governed by a confidentiality agreement between the principal investigator and Proxima Research LLC for the study period from 2010 to 2022.

### Variables of interest

The company Proxima Research is responsible for collecting, processing, and organizing both pharmacy sales and prescription data, which are systematically reviewed and categorized using the Anatomical Therapeutic Chemical classification system. In this study, the primary outcome was psychopharmacological medication utilisation, operationalized as the annual number of packages dispensed from community pharmacies (pharmacy sales) within ATC group N (Nervous system). This outcome reflects medicines actually dispensed to patients in the outpatient retail sector rather than the number of prescriptions issued.

For the trend analyses based on Sell-Out data, we focused specifically on psychopharmacological agents and selected the following third-level ATC subgroups: N05A Antipsychotics, N05B Anxiolytics, N05C Hypnotics and sedatives, N06A Antidepressants, N06B Psychostimulants, agents used for ADHD and nootropics. The analysis compared trends across these key psychopharmacological subgroups. Additionally, we examined specific medications within these subgroups, focusing on the most frequently dispensed medications.

For the descriptive analysis of prescription counts by sex and age, we used RxTest data for 2015–2022 and included prescriptions in ATC groups N04 Anti-Parkinson drugs, N05 Psycholeptics, N06 Psychoanaleptics, and N07 Other nervous system drugs. Although N04 medications are primarily neurological, they are frequently co-prescribed in patients with severe mental disorders; we therefore included N04 in descriptive analyses only.

### Analysis

Our analysis compared trends in sales of psychopharmacological subgroups, focusing on antipsychotics (N05A), anxiolytics (N05B), hypnotics/sedatives (N05C), antidepressants (N06A), and psychostimulants (N06B) from 2010 to 2022, as these subgroups were most relevant to the diagnostic groups of interest. The primary outcome variable was annual pharmacy sales, operationalized as the annual number of packages dispensed from pharmacies (Sell-Out data) analyzed at the ATC subgroup and by active substance (international nonproprietary name, INN) to describe patterns and trends over time.

To provide demographic context, we also reviewed prescription volume data from 2015 to 2022, categorized by patient sex and age group. This analysis focused specifically on medications used for psychiatric conditions, allowing us to describe general trends in prescription activity without assessing individual prescribing behaviors or clinical decision-making.

We used Stata SE 18 (StataCorp. 2023. *Stata Statistical Software: Release 18.* College Station, TX: StataCorp LLC.) to summarize psychopharmacological medication packages by aggregating sales and usage data across subgroups.

Joinpoint regression was used to analyze annual trends. Inspection of the time-series plots indicated that annual trends were not well represented by a single linear function over the entire study period. We therefore used joinpoint regression to allow for changes in slope over time and to identify potential change points in a data-driven manner. Interrupted time series or difference-in-differences approaches were not applied because there was no single pre-specified intervention date, and the 2010–2022 period includes multiple major events that do not fit a simple before-after framework. For each psychopharmacological subgroup, we modelled the natural logarithm of the annual number of dispensed packages, ln(y), as a function of calendar year. Given the limited number of time points (13 annual observations), we allowed between 0 and 2 joinpoints (i.e., up to three linear segments per time series).

The number and location of joinpoints were selected using a permutation test with an overall significance level of 0.05, as implemented in the Joinpoint Regression Program, version 5.4.0 (National Cancer Institute: *Joinpoint Regression Program*,* Version 5.4.0*. Statistical Research and Applications Branch, Surveillance Research Program, National Cancer Institute, Bethesda, MD, USA).

For each subgroup of psychopharmacological medications, we estimated the Annual Percent Change (APC) and the Average Annual Percent Change (AAPC) with corresponding 95% confidence intervals (CIs). APC and AAPC estimates, together with their 95% CIs, were obtained using the empirical quantile method with 5,001 resamples. Trends were considered statistically significant if the 95% CI for the APC or AAPC did not include zero (α = 0.05).

## Results

### Descriptive characteristics of the study population

The data presented in Table 1 provide a summary of prescription counts within the study population from 2015 to 2022 for N04 Anti-Parkinson drugs, N05 Psycholeptics, N06 Psychoanaleptics, and N07 Other nervous system drugs. The table illustrates the total number of prescriptions categorized by sex. Additional file 1 presents how prescriptions were distributed among males and females across predefined age groups (1–18, 18–30, 30–40, 40–50, 50–70, and over 70 years). For instance, the category 18–30 years includes individuals aged 18–29 years, but not those who have turned 30. The highest number of prescriptions with unspecified sex was observed in 2015, with no unspecified sex recorded from 2018.

Overall, the data from Table 1 underscore the dynamic nature of prescription patterns for the nervous system in specific classes. In 2015, the total number of prescriptions was 9,249,253, and it remained relatively stable until 2017, after which a gradual decline was observed. Between 2015 and 2020, there was a marked reduction in the total number of prescriptions issued, reaching 5,517,520 in 2020. Although there was a slight increase in 2021, the total number of prescriptions in 2022 (5,904,554) remained clearly below the 2015 level. This indicates an overall reduction in prescriptions issued over the period under review.

Across all years, females consistently had a higher number of prescriptions compared to males. In 2015, females accounted for 4,194,052 prescriptions, whereas males had 3,878,414 prescriptions. This trend persisted over subsequent years, although both females and males experienced a decline in prescriptions over time.

**Table 1:** Total number of prescriptions from 2015 to 2022 for N04 Anti-Parkinson drugs, N05 Psycholeptics, N06 Psychoanaleptics, and N07 Other nervous system drugs

Year	2015	2016	2017	2018	2019	2020	2021	2022
Total number of prescriptions	9 249 253	9 299 937	8 966 241	8 674 708	8 286 557	5 517 520	6 435 978	5 904 554
**Sex**:								
Male	3 878 414	4 196 694	4 186 269	4 122 876	3 911 380	2 567 429	2 999 250	2 793 475
n (%)	41.9	45.1	46.7	47.5	47.2	46.5	46.6	47.3
Female	4 194 052	4 639 451	4 775 488	4 551 832	4 375 177	2 950 091	3 436 728	3 111 079
n (%)	45.4	49.8	53.3	52.5	52.8	53.5	53.4	52.7
Unspecified	1 176 787	463 792	4 484					
n (%)	12.7	5.1	0.05					

Source: PharmXplorer database^®^ Proxima Research LLC, 2009–2023

### Psychopharmacological drug consumption

#### Descriptive statistics for the overall consumption of selected nervous-system drugs from 2010 to 2022

**Fig. 1:**
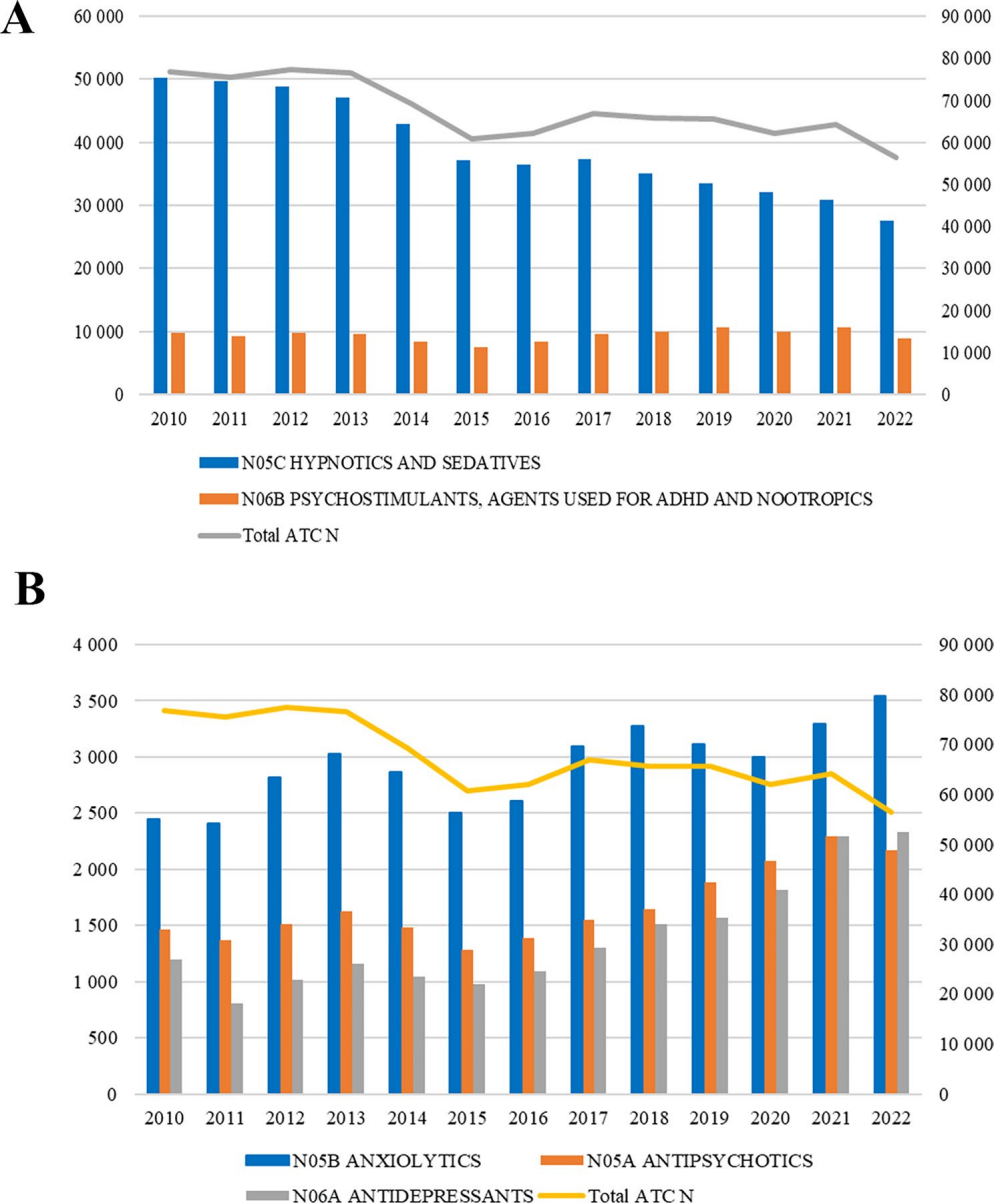
**A**: Annual number of packages dispensed from pharmacies for N05C hypnotics and sedatives, and N06B psychostimulants, agents used for ADHD and nootropics (bars; left y-axis), and all nervous-system drugs combined (Total ATC N; ATC group N03-07; line; right y-axis) in Ukraine, 2010–2022 (packages x 1.000). Source: PharmXplorer database^®^ Proxima Research LLC, 2009–2023. **B**: Annual number of packages dispensed from pharmacies for N05B anxiolytics, N05A antipsychotics, and N06A antidepressants (bars; left y-axis), and all nervous-system drugs combined (Total ATC N; ATC group N03-07; line; right y-axis) in Ukraine, 2010–2022 (packages x 1.000). Source: PharmXplorer database^®^ Proxima Research LLC, 2009–2023

Figure 1A and B show trends in the annual number of packages of psychopharmacological medications dispensed from pharmacies in Ukraine from 2010 to 2022. Five psychopharmacological subgroups (N05A, N05B, N05C, N06A, N06B) are plotted alongside a “Total ATC N” series, which summarizes the combined utilisation of N03-N07 nervous-system medications in the outpatient retail sector. Key findings reveal a consistent decline in hypnotics and sedatives (N05C), an upward trend in anxiolytics (N05B) and antidepressants (N06A), antipsychotics (N05A), and minimal changes in psychostimulants (N06B). Detailed numerical data are provided in Additional file 2 and Additional file 3.

Overall, total ATC N sales fluctuated over the period, with the highest observed in the early years (76.8 million packages in 2010 and 77.4 million in 2012), followed by an overall decline to 56.4 million packages in 2022. However, subgroup-specific trends varied:

- For N05C hypnotics and sedatives, utilisation showed a steady decrease, from 50.2 million packages in 2010 to 27.6 million in 2022.
- For N06B psychostimulants, agents used for ADHD and nootropics remained stable with a range from 7.4 million packages in 2015 to 10.7 million in 2021.
- Also, N05B anxiolytics, utilisation fluctuated but showed an overall increase from 2.4 million packages in 2010 to above 3.5 million in 2022.
- N05A antipsychotics, this group increased from about 1.4 million packages in 2010 to 2.3 million in 2022.
- Similarly, for N06A antidepressants, there was a steady increase in consumption, rising from 1.2 million in 2010 to 2.3 million in 2022.

#### Psychopharmacological medication use from 2010 to 2022

The consolidated joinpoint regression results are summarized in Table 2 below. Detailed results are provided in Additional files 4–13, available as supplementary material.

For hypnotics and sedatives (N05C), the joinpoint regression did not identify any change in trend; a single declining segment was fitted for 2010–2022. Over this period, use decreased steadily with an APC of -4.9% (95% CI -5.5% to -4.2%; *p* < 0.001). The AAPC for the full period was also − 4.9% (95% CI -5.5% to -4.2%; *p* < 0.001), indicating a sustained long-term decline. Among hypnotics and sedatives, barbiturates showed the highest sales in 2010, but their usage steadily declined, with packages decreasing from 25.2 million in 2010 to 10.5 million in 2022.

For psychostimulants (N06B), joinpoint regression identified two changes in trend, in 2015 and 2019. Between 2010 and 2015, the number of packages decreased significantly (APC − 4.9%; 95% CI -13.8% to -1.5%; *p* = 0.014). From 2015 to 2019, consumption increased (APC 7.8%; 95% CI 3.1% to 15.2%; *p* = 0.018). After 2019, use declined again, but this trend was not statistically significant (APC − 4.3%; 95% CI -14.9% to 1.0%; *p* = 0.096). Over the full study period, the AAPC was − 0.7% (95% CI -2.3% to 0.5%; *p* = 0.212), indicating no significant long-term change in overall psychostimulant consumption. The leading drug in this category, phenibut, exhibited a steady increase, peaking at around 3.4 million packages in 2022.

On the other hand, the analysis of anxiolytics, antipsychotics, and antidepressants indicated increased use over time. For anxiolytics (N05B), the joinpoint regression analysis did not detect any change in trend; a single increasing segment was fitted for 2010–2022. Over this period, anxiolytic use increased significantly, with an APC of 2.6% (95% CI 0.7% to 4.4%; *p* = 0.006). The AAPC for the whole period was also 2.6% (95% CI 0.7% to 4.4%; *p* = 0.006), indicating a sustained long-term rise in use. The leading drug in this category, gidazepam, exhibited consistent growth, increasing from around 1.7 million packages in 2010 to above 3 million packages in 2022.

For antipsychotics (N05A), joinpoint regression analysis identified one change in trend in 2016. Between 2010 and 2016, there was no statistically significant change in use. APC was − 0.9% (95% CI -7.3% to 2.2%; *p* = 0.545). From 2016 to 2022, antipsychotic use increased markedly, with an APC of 8.9% (95% CI 5.6% to 16.2%; *p* < 0.001). Over the full study period, the AAPC was 3.9% (95% CI 2.5% to 5.3%; *p* < 0.001), indicating a sustained long-term rise in antipsychotic consumption. The leading medicine in the group was sulpiride, rising from 310 thousand to 455 thousand packages. Quetiapine showed the steepest growth, from only 5 thousand packages to 448 thousand in 2022, becoming comparable to sulpiride by the end of the period.

For antidepressants (N06A), joinpoint regression identified one change in trend in 2015. Between 2010 and 2015, antidepressant use showed no statistically significant trend, APC − 0.7% (95% CI -15.2% to 5.6%; *p* = 0.734). From 2015 to 2022, consumption increased with an APC of 13.3% (95% CI 9.2% to 27.8%; *p* = 0.002). Over the full study period, the AAPC was 7.2% (95% CI 5.0% to 9.8%; *p* < 0.001), indicating a sustained long-term rise in antidepressant use. Notably, amitriptyline was the most used antidepressant in 2010, whereas escitalopram, a selective serotonin reuptake inhibitor (SSRI), gradually became the dominant medication, increasing from 33 thousand packages in 2010 to above 515 thousand in 2022.

**Table 2:** Joinpoint regression results (Annual percent change (APC), and Average Annual Percent Change (AAPC)) for the annual number of dispensed packages of selected psychopharmacological drug groups, Ukraine, 2010 to 2022

ATC group	Trend measure	Years	% change per year	95% CI	*P*-value
N05C Hypnotics and Sedatives	APC	2010–2022	-4.9*	-5.5; -4.2	< 0.001
	**AAPC**,** overall**	2010–2022	-4.9*	-5.5; -4.2	< 0.001
N06B Psychostimulants (ADHD, nootropics)	APC, segment 1	2010–2015	-4.9*	-13.8; -1.5	0.014
	APC, segment 2	2015–2019	7.8*	3.1; 15.2	0.018
	APC, segment 3	2019–2022	-4.3	-14.9; 1	0.096
	**AAPC**,** overall**	2010–2022	-0.7	-2.3; 0.5	0.212
N05B Anxiolytics	APC	2010–2022	2.6*	0.7; 4.4	0.006
	**AAPC**,** overall**	2010–2022	2.6*	0.7; 4.4	0.006
N05A Antipsychotics	APC, segment 1	2010–2016	-0.9	-7.3; 2.2	0.545
	APC, segment 2	2016–2022	8.9*	5.6; 16.2	< 0.001
	**AAPC**,** overall**	2010–2022	3.9*	2.5; 5.3	< 0.001
N06A Antidepressants	APC, segment 1	2010–2015	-0.7	-15.2; 5.6	0.734
	APC, segment 2	2015–2022	13.3*	9.2; 27.8	0.002
	**AAPC**,** overall**	2010–2022	7.2*	5.0; 9.8	< 0.001

*Indicates that the Annual Percent Change (APC) or Average Annual Percent Change (AAPC) is significantly different from zero at the α = 0.05 level

## Discussion

This registry-based study examined trends in psychopharmacological medication use in Ukraine from 2010 to 2022. Data were drawn from the PharmXplorer database^®^, and analyses focused on ATC group N (Nervous system) medications, particularly hypnotics and sedatives (N05C), psychostimulants (N06B), anxiolytics (N05B), antipsychotics (N05A), and antidepressants (N06A). Findings revealed changes in dispensed packages (Sell-Out, 2010–2022) and a decline in prescription counts (RxTest, 2015–2022), together with shifts within drug classes, which may reflect broader health system changes [4, 5].

A notable decline in prescriptions occurred from 2019 to 2020, coinciding with the COVID-19 pandemic, and again in 2022 following the onset of the full-scale war. This reduction may reflect disruptions to healthcare access and widespread displacement, rather than a reduction in mental health needs.

In RxTest, prescription rates were consistently higher among women, in line with known global patterns of mood and anxiety disorders, as well as gender differences in healthcare-seeking behavior [3].

Dispensed packages (Sell-Out) peaked in 2010 and 2012, followed by a decline after 2014. This trend may be influenced by broader contextual factors, including the annexation of Crimea and the abandonment of pharmaceutical substances previously sourced from Russia [16, 17]. After a period of relative stability, further decreases were observed in 2020 and 2022, which could be associated with disruptions caused by the COVID-19 pandemic and the onset of the full-scale war [18, 19].

The use of N05C hypnotics and sedatives showed a steady decline over the study period. Despite this reduction, barbiturate-based combinations, such as Corvalol, remain widely used, particularly among older adults. Ukrainian regulations permit the over-the-counter sale of certain phenobarbital-containing products, provided they fall within defined dosage limits [20]. At the same time, there has been a growing interest in herbal sedatives like valerian root, suggesting a gradual shift toward safer, non-prescription alternatives and a broader trend in favor of evidence-based and integrative approaches to managing sleep disturbances [21, 22]. European data show a more heterogeneous picture. In Spain, a nationwide study covering 2005–2022 reported an overall increase in hypnotic and sedative use, with particularly high prevalence among middle-aged and older women. However, that analysis combined N05B anxiolytics and N05C hypnotics and sedatives, making it difficult to isolate group N05C specifically [23]. In Portugal, a national time-series analysis of outpatient psychotropic use from 2000 to 2018 found relatively low levels of sedatives and hypnotics use compared with other psychotropic classes [24]. Age-stratified data from Scandinavia indicate a shift in the composition of hypnotic and sedative use: among 5-24-year-olds in Norway, Denmark, and Sweden (2012–2018), melatonin became the most commonly used ‘hypnotic’ or sleep-promoting drug [25]. Taken together, these observations suggest that Ukraine may differ from some European settings. Our data clearly show a marked decline in N05C use in Ukraine, while barbiturate-containing products still account for an important share of use.

Over the 13-year study period, the overall utilisation of N06B psychostimulants and nootropic agents remained relatively stable. However, notable shifts were observed within the group in terms of the most frequently used substances. In 2010, piracetam held the leading position. A significant shift occurred in 2017, when phenibut became the dominant product in this group. In 2022, around 3.4 million packages were sold. Chemically known as β-phenyl-γ-aminobutyric acid, phenibut is a derivative of the neurotransmitter GABA and was first developed in the Soviet Union in the 1960s [26]. In Ukraine, it is approved for a wide range of conditions [27]. Although initially perceived as effective in reducing stress and anxiety, phenibut carries a well-documented risk of dependence and withdrawal symptoms with prolonged use [28]. The situation is further complicated by the fact that phenibut is available over the counter in Ukraine and is widely promoted via television and online platforms. As a result, many consumers may not be aware of the potential harms, including tolerance and addiction.

This pattern contrasts sharply with recent European data, where N06B utilisation is increased by ADHD-specific medicines rather than nootropic agents. In a study of 28 European countries consumption of ADHD medications (e.g. methylphenidate, lisdexamfetamine) rose from 1.85 to 3.43 defined daily doses (DDD) per 1,000 inhabitants per day between 2014 and 2022 with particularly marked increases after the COVID-19 pandemic and especially high use in Scandinavian and Western European countries [29]. Direct quantitative comparison with these European estimates is not possible because our data are based on dispensed packages rather than DDD per 1,000 inhabitants per day. However, the overall pattern of N06B use clearly differs. These differences may reflect underdiagnosis of ADHD in Ukraine and limited access to specialist child and adolescent mental health services. Together, these findings suggest a possible unmet need for evidence-based ADHD diagnosis and treatment in Ukraine.

Trends in the use of N05B anxiolytics have also increased. The most commonly used anxiolytic on the Ukrainian market is gidazepam, a benzodiazepine derivative developed during the Soviet era [30]. The popularity of gidazepam is likely influenced by regulatory gaps, since it is not listed as a controlled substance internationally, it remains largely unregulated within the Ukrainian system [31]. The highest level of its consumption was recorded in 2022, with over 3 million packages, potentially reflecting heightened levels of population stress and anxiety following the onset of the full-scale war. In contrast, recent European studies have reported a general decline in anxiolytic consumption between 2012 and 2021, with significant downward trends in most countries, and only increases in Latvia and Spain [32]. Similar patterns are seen in Scandinavia, where a prescription database study from Norway, Denmark, and Sweden reported a marked decline in anxiolytic use between 2006 and 2021, with prevalence falling most steeply in Denmark [33]. Thus, the upward trend in anxiolytic use in Ukraine is driven largely by gidazepam. This differs from broader European and Scandinavian patterns and raises concerns regarding medication safety, dependence, and public awareness. The availability of this product, combined with limited knowledge among patients about their potential risks, underscores the urgent need for improved pharmacovigilance, prescriber education, and public health campaigns to promote the rational and safe use of anxiolytic medications.

The use of N05A antipsychotics showed a moderate but sustained increase over the study period. Throughout the period, sulpiride remained the single most dispensed antipsychotic; however, the steepest growth was observed in quetiapine. One explanation for the rise in quetiapine use is its inclusion in the national “Affordable Medicines” reimbursement programme, so patients can receive it free of charge or with a small co-payment instead of the full price [8]. In contrast, sulpiride, although previously included in the National List of Essential Medicines, was later removed, which may have attenuated its growth relative to quetiapine [34]. Patterns of antipsychotic use in Ukraine are consistent with recent European data. A multi-database study from the DARWIN EU initiative reported variations in overall antipsychotic utilisation, but a clear shift towards second-generation (atypical) agents, particularly quetiapine, olanzapine, and risperidone. The increase in atypical antipsychotic use in our study, and especially for quetiapine, is therefore in line with the European trend. Importantly, the DARWIN EU report also showed that a considerable proportion of antipsychotic use occurred in people without a record of a psychotic disorder diagnosis, suggesting frequent prescribing for non-psychotic conditions (depression, insomnia, other sleep disorders). Our data do not contain patient-level diagnostic information linked to prescriptions or dispensed packages, so we cannot distinguish on-label from off-label use in Ukraine. However, the increasing volumes of atypical antipsychotics, particularly quetiapine, may raise questions about the indications for antipsychotic use for non-psychotic conditions [35].

The use of N06A antidepressants showed growth: from about 1.2 million packages in 2010 to over 2.3 million in 2022, with the highest use recorded during the first year of the full-scale war. This upward trend may reflect both a growing demand for mental health treatment and a shift in prescription patterns aligned with clinical guidelines. A notable change occurred in the choice of leading antidepressants. In 2010, amitriptyline - a tricyclic antidepressant - was the most commonly used agent. However, by 2022, escitalopram, a selective serotonin reuptake inhibitor (SSRI), had become the most prescribed antidepressant. The earlier dominance of amitriptyline may have been influenced by its inclusion in Ukraine’s National List of Essential Medicines [34], which guarantees basic treatment access for the population and emphasizes cost-effective options. In contrast, the rising preference for escitalopram may be attributed to ongoing mental health reform efforts, including the integration of the WHO’s mhGAP program. Training and support for primary care physicians have likely contributed to improved recognition of mental health conditions and greater use of modern, evidence-based pharmacological strategies [36, 37].

Our findings are broadly consistent with data from other European countries. In a recent time-series analysis of 14 European countries, antidepressant consumption (measured as defined daily doses per 1,000 inhabitants per day) increased in almost all countries between 2012 and 2021 [32]. Similar patterns were observed in Scandinavian countries, where national prescription registries documented a rise in SSRI use across all age groups [33]. Although these studies used DDD-based measures, whereas our analysis is based on dispensed packages, the direction of change is comparable and supports a general trend towards increasing antidepressant use across Europe and Scandinavia.

### Implications for mental health policy and practice

The increasing use of anxiolytic medications in Ukraine, particularly gidazepam, raises important concerns regarding potential misuse and dependence. Although gidazepam is a benzodiazepine derivative, it is not scheduled under international control, which limits regulatory oversight and poses significant challenges for monitoring and restricting its use. Additionally, phenibut, another over-the-counter product, remains widely accessible despite its well-documented risk of abuse and dependence.

These trends highlight the need for strengthened pharmacovigilance, regulatory frameworks, and public education to promote the rational use of anxiolytics and over-the-counter products such as phenibut and mitigate the associated health risks. Enhanced training for prescribers and clearer labeling requirements may also support safer use and improve mental health care outcomes.

### Strengths and limitations

This study has several limitations. First, some components of the PharmXplorer^®^ system (e.g. prescription monitoring in RxTest) may rely partly on doctors’ self-reports via online surveys or telephone interviews, which introduces a risk of recall bias or reporting bias in prescription information. Second, the PharmXplorer database^®^ is sponsored by pharmaceutical companies, which may raise concerns about potential conflicts of interest. However, the database is used for commercial decision-making purposes, so participating companies have a strong incentive to ensure that data collection and processing are objective and of high quality.

Third, our utilisation measure was based on pharmacy sales data from the retail sector (Sell-Out project), expressed as the annual number of dispensed packages, rather than individual-level treatment records. These data capture outpatient medicine use and do not include medicines administered exclusively in hospitals or other inpatient settings. Moreover, pharmaceutical sales registers do not perfectly reflect actual medication use: we could not determine whether all dispensed medicines were taken as prescribed, nor could we account for prescriptions that were issued (captured in RxTest) but never collected at the pharmacy. However, for prescription-only psychopharmacological medicines, most purchases in community pharmacies are typically based on a doctor’s prescription. Therefore, Sell-Out data reflect the volume of medicines dispensed from community pharmacies in the outpatient setting, although they remain subject to the limitations described above. Furthermore, non-adherence to prescribed regimens by patients may introduce inaccuracies, potentially skewing the interpretation of usage patterns.

Finally, information on DDD per package was not available. We were therefore unable to express utilisation in DDDs (e.g., DDD per 1,000 inhabitants per day) or to adjust for possible changes in strength or pack size over time. As a result, our estimates reflect trends in the volume of packages dispensed in the outpatient retail sector, but may not fully capture changes in dose intensity or duration of treatment.

This study also does not include information on patient outcomes, such as medication effectiveness, side effects, or long-term adherence. As a result, conclusions about clinical impact remain limited. Moreover, the dataset is restricted to government-controlled (unoccupied) regions and does not fully capture healthcare access or treatment patterns in conflict-affected areas. In addition, PharmXplorer^®^ data collection is concentrated in major urban centres, and we could not assess geographic access directly. Dispensed packages may be partially captured in pharmacies located in nearby towns or urban centres, and we were unable to evaluate utilisation patterns specifically in rural settings. These geographical limitations highlight the importance of contextualizing the findings and recognizing potential disparities in mental health care during the war and other major disruptions.

Despite these limitations, the study provides valuable contributions to the understanding of psychopharmacological medication use in Ukraine from 2010 to 2022. To our knowledge, representative studies specifically focused on psychopharmacological medication use in Ukraine are limited. One of the few available analyses is the “Comparative analysis of the consumption of antidepressants in Ukraine, Estonia, and Norway” [38], which offers valuable insight but does not fully capture trends. Other existing research, including recent publications such as “Mental health in Ukraine in 2023”, “Editorial: Measuring and Buffering the Mental Health Impact of the War in Ukraine in Young People”, etc., mainly concentrates on the psychological consequences of war and health system resilience [39, 40]. While these studies are important for understanding broader mental health challenges, they do not explore trends in the use of psychopharmacological treatments. This underscores the unique contribution of our study, which provides a long-term perspective on prescribing practices and consumption dynamics across multiple psychotropic drug classes in Ukraine.

## Conclusion

In conclusion, our comprehensive analysis of psychopharmacological medication trends in Ukraine from 2010 to 2022 provides insight into the complexity of mental health care in the country. Despite challenges, including limited literature comparable in scope to our study, we observed changes in prescription volumes that occurred during major socio-political events and healthcare reforms. Notably, psychopharmacological medication prescriptions declined between 2015 and 2022, with marked decreases in 2020 and 2022 during the COVID-19 pandemic and the onset of the full-scale war in Ukraine. These events likely affected healthcare delivery and contributed to population displacement, creating additional challenges for mental healthcare provision. In parallel, trends in pharmacy-dispensed packages during 2010–2022 within specific ATC classifications indicate shifts over time, including declining use of hypnotics and sedatives and increasing use of antipsychotics, antidepressants, and anxiolytics.

Study limitations warrant cautious interpretation of trends within each data source and underscore the need for further research to contextualize findings within the broader landscape of mental health policy and clinical practice. In summary, this study highlights the importance of strengthening the integration of mental health services into primary care, enhancing regulatory measures to support safe medication practices, and addressing the complex challenges facing the Ukrainian healthcare system. By understanding factors associated with changes in prescription and dispensing trends, policymakers, healthcare providers, and stakeholders can work together to support equitable access to quality mental health care for all Ukrainians.

## Supplementary Information

Below is the link to the electronic supplementary material.

Supplementary Material 1: Additional file 1: Total prescriptions by year, gender, and age categorization from 2015 to 2022

Supplementary Material 2: Additional file 2: Descriptive table for overall consumption of ATC N03-N07 nervous-system drugs from 2010 to 2022

Supplementary Material 3: Additional file 3: Descriptive table for overall consumption of a specific group of drugs from 2010 to 2022

Supplementary Material 4: Additional file 4: The most frequently dispensed hypnotic and sedative medications (N05C), categorized by 5th level ATC code and measured in packages from 2010 to 2022

Supplementary Material 5: Additional file 5: The most frequently dispensed psychostimulant medications (N06B), categorized by 5th level ATC code and measured in packages from 2010 to 2022

Supplementary Material 6: Additional file 6: The most frequently dispensed anxiolytic medications (N05B), categorized by the 5th level ATC code and measured in packages from 2010 to 2022

Supplementary Material 7: Additional file 7: The most frequently dispensed antipsychotic medications (N05A), categorized by the 5th level ATC code and measured in packages from 2010 to 2022

Supplementary Material 8: Additional file 8: The most frequently dispensed antidepressant medications (N06A), categorized by 5th level ATC code and measured in packages from 2010 to 2022

Supplementary Material 9: Additional file 9: Joinpoint regression plot for hypnotics and sedatives (N05C), 2010–2022

Supplementary Material 10: Additional file 10: Joinpoint regression plot for psychostimulants, agents used for ADHD, and nootropics (N06B), 2010–2022

Supplementary Material 11: Additional file 11: Joinpoint regression plot for anxiolytics (N05B), 2010–2022

Supplementary Material 12: Additional file 12: Joinpoint regression plot for antipsychotics (N05A), 2010–2022

Supplementary Material 13: Additional file 13: Joinpoint regression plot for antidepressants (N06A), 2010–2022

## Data Availability

VZ got access to the database upon request from Proxima Research LLC, holding the database ownership. VZ had permission to conduct independent research, but no third-party sharing. Access to the data and data agreements must be addressed to Proxima Research LLC.

## Abbreviations

AAPC: Average Annual Percent Change
ADHD: Attention-Deficit/Hyperactivity Disorder
APC: Annual Percent Change
ATC: Anatomical Therapeutic Chemical
DALY: Disability Adjusted Life Year
DDD: Defined Daily Dose
ICD: International Classification of Diseases
INN: International Nonproprietary Name
LLC: Limited Liability Company
mhGAP: Mental Health Gap Action Programme
NHSU: National Health Service of Ukraine
PTSD: Post-Traumatic Stress Disorder
REK: Regional Committees for Medical and Health Research Ethics in Norway
SSRI: Selective Serotonin Reuptake Inhibitors
WHO: World Health Organization

## References

[1] World Health Organization. Global burden of mental disorders and the need for a comprehensive, coordinated response from health and social sectors at the country level: report by the Secretariat. Executive Board 130th session, provisional agenda item 6.2, EB130/9. 1 December 2011. Available from: https://apps.who.int/gb/ebwha/pdf_files/eb130/b130_9-en.pdf. Accessed 30 September 2023.

[2] Hock RS, Or F, Kolappa K, Burkey MD, Surkan PJ, Eaton WW. A new resolution for global mental health. Lancet. 2012;379(9824):1367–8. 10.1016/S0140-6736(12)60243-8. 22500865 10.1016/S0140-6736(12)60243-8 PMC4767178

[3] World Health Organization. World mental health report: transforming mental health for all. Geneva: World Health Organization. 2022. Available from: https://www.who.int/publications/i/item/9789240049338. Accessed 30 September 2023.

[4] Weissbecker I, Khan O, Kondakova N, Poole LA, Cohen J. Mental health in transition: assessment and guidance for strengthening integration of mental health into primary health care and community-based service platforms in Ukraine. Washington, DC: World Bank Group; 2017 [cited 2023 Oct 30]. Available from: https://documents1.worldbank.org/curated/en/310711509516280173/pdf/120767-WP-Revised-WBGUkraineMentalHealthFINALwebvpdfnov.pdf.

[5] Cabinet of Ministers of Ukraine. Approval of the Concept of Mental Health Care Development in Ukraine for the Period up to 2030. Order No. 1018-r, 27 Dec 2017. Available from: https://zakon.rada.gov.ua/laws/show/1018-2017-%D1%80#Text. Accessed 30 October 2023.

[6] Ministry of Health of Ukraine. National Health Service of Ukraine has been operating for 5 years. 30. March 2023. Available from: https://moz.gov.ua/en/national-health-service-of-ukraine-has-been-operating-for-5-years. Accessed 5 November 2025.

[7] Ministry of Health of Ukraine. Electronic health care system. Available from: https://moz.gov.ua/uk/elektronna-sistema-ohoroni-zdorovya. Accessed 5 November 2025.

[8] Ministry of Health of Ukraine. Affordable Medicines programme: which medicines can patients receive free of charge? 30 May 2023. Available from: https://moz.gov.ua/uk/programa-dostupni-liki-jaki-liki-pacient-mozhe-otrimati-bezoplatno. Accessed 27 November 2025.

[9] Cabinet of Ministers of Ukraine. National Health Service of Ukraine dashboards reopened: necessary information can be obtained instantly. 28 December 2022. Available from: https://www.kmu.gov.ua/news/dashbordy-nszu-znovu-u-vidkrytomu-dostupi-neobkhidnu-informatsiiu-mozhna-otrymaty-myttievo. Accessed 27 November 2025.

[10] Charlson F, van Ommeren M, Flaxman A, Cornett J, Whiteford H, Saxena S. New WHO prevalence estimates of mental disorders in conflict settings: a systematic review and meta-analysis. Lancet. 2019;394(10194):240–8. 10.1016/S0140-6736(19)30934-1. 31200992 10.1016/S0140-6736(19)30934-1 PMC6657025

[11] Cardozo BL, Kaiser R, Gotway CA, Agani F. Mental health, social functioning, and feelings of hatred and revenge of Kosovar Albanians one year after the war in Kosovo. J Trauma Stress. 2003;16(4):351–60. 10.1023/A:1024413918346. 12895018 10.1023/A:1024413918346

[12] Cardozo BL, Vergara A, Agani F, Gotway CA. Mental health, social functioning, and attitudes of Kosovar Albanians following the war in Kosovo. JAMA. 2000;284(5):569–77. 10.1001/jama.284.5.569. 10918702 10.1001/jama.284.5.569

[13] Cardozo BL, Bilukha OO, Crawford CAG, Shaikh I, Wolfe MI, Gerber ML, et al. Mental health, social functioning, and disability in postwar Afghanistan. JAMA. 2004;292(5):575–84. 10.1001/jama.292.5.575. 15292083 10.1001/jama.292.5.575

[14] Scholte WF, Olff M, Ventevogel P, de Vries GJ, Jansveld E, Cardozo BL, et al. Mental health symptoms following war and repression in Eastern Afghanistan. JAMA. 2004;292(5):585–93. 10.1001/jama.292.5.585. 15292084 10.1001/jama.292.5.585

[15] Kakaje A, Al Zohbi R, Hosam Aldeen O, et al. Mental disorder and PTSD in Syria during wartime: a nationwide crisis. BMC Psychiatry. 2021;21:2. 10.1186/s12888-020-03002-3. 33388026 10.1186/s12888-020-03002-3 PMC7778805

[16] Treisman D. Why Putin took Crimea: the gambler in the Kremlin. Foreign Affairs. 2016 May/Jun [cited 2023 Nov 25]. Available from: https://www.foreignaffairs.com/articles/ukraine/2016-04-18/why-russian-president-putin-took-crimea-from-ukraine.

[17] Apteka online. Sanctions against Russia: impact on the Ukrainian pharmaceutical market. Apteka.ua. 2014 Sep 24. Available from: https://www.apteka.ua/article/307450. Accessed 18 October 2023.

[18] Oros MM, Pryhoda NF. Anxiety and COVID-19. Journal of Neurology named after B. M. Mankovskyi. 2021;9(4):30–32 [cited 2023 Oct 18]. Available from: http://nbuv.gov.ua/UJRN/jorn_2021_9_4_6.

[19] International Organization for Migration (IOM). Ukraine — conditions of return assessment factsheet — round 4 (July–August 2023). IOM UN Migration; 2023. Available from: https://dtm.iom.int/reports/ukraine-conditions-return-assessment-factsheet-round-4-july-august-2023. Accessed 22 September 2023.

[20] Ministry of Health of Ukraine. Order No. 210 dated 14 May 2003 on approval of the criteria for classifying narcotic (psychotropic) drugs containing small amounts of narcotic drugs or psychotropic substances and precursors as over-the-counter drugs, and the list of these drugs. Available from: https://www.dec.gov.ua/materials/shhodo-obigu-otrujnih-ta-silnodiyuchih-lz/. Accessed 15 December 2023.

[21] Office of Dietary Supplements. Valerian: fact sheet for health professionals. National Institutes of Health. Available from: https://ods.od.nih.gov/factsheets/Valerian-HealthProfessional/. Accessed 15 January 2024.

[22] European Medicines Agency. Valerianae radix – herbal medicinal product. Available from: https://www.ema.europa.eu/en/medicines/herbal/valerianae-radix. Accessed 15 January 2024.

[23] Mourino N, Teijeiro A, Guerra-Tort C, Rey-Brandariz J, Candal-Pedreira C, Martín-Gisbert L, et al. Consumption of hypnosedatives in spain: characterization and time trends, 2005–2022. Gac Sanit. 2024;38:102433. 10.1016/j.gaceta.2024.102433. 39602859 10.1016/j.gaceta.2024.102433

[24] Estrela M, Herdeiro MT, Ferreira PL, Roque F. The use of antidepressants, anxiolytics, sedatives and hypnotics in europe: focusing on mental health care in Portugal and prescribing in older patients. Int J Environ Res Public Health. 2020;17(22):8612. 10.3390/ijerph17228612. 33228203 10.3390/ijerph17228612 PMC7699589

[25] Wesselhoeft R, Rasmussen L, Jensen PB, Jennum PJ, Skurtveit S, Hartz I, et al. Use of hypnotic drugs among children, adolescents, and young adults in Scandinavia. Acta Psychiatr Scand. 2021;144(2):100–12. 10.1111/acps.13329. 34021908 10.1111/acps.13329

[26] Lapin I. Phenibut (beta-phenyl-GABA): a tranquilizer and nootropic drug. CNS Drug Rev. 2001 Winter;7(4):471–81. 10.1111/j.1527-3458.2001.tb00211.x. 10.1111/j.1527-3458.2001.tb00211.x PMC6494145 11830761

[27] Ministry of Health of Ukraine. Pharmaceutical management. State Expert Center of the Ministry of Health of Ukraine. State register of medicines of Ukraine. Available from: http://www.drlz.com.ua/ibp/ddsite.nsf/all/shlz1?opendocument%26stype=1BDDA06A96688BC6C2258AE600376814. Accessed 18 January 2024.

[28] Feldman R, Autry B, Dukes J, Lofy T, Marchetti G, Patt A, et al. A systematic review of phenibut withdrawal focusing on complications, therapeutic approaches, and single substance versus polysubstance withdrawal. Clin Toxicol (Phila). 2023;61(11):941–51. 10.1080/15563650.2023.2248325. 38112312 10.1080/15563650.2023.2285702

[29] Gimbach S, Vogel D, Fried R, Faraone SV, Banaschewski T, Buitelaar J, et al. ADHD medicine consumption in Europe after COVID-19: catch-up or trend change? BMC Psychiatry. 2024;24:112. 10.1186/s12888-024-05505-9. 38336744 10.1186/s12888-024-05505-9 PMC10854136

[30] Zhabenko O, Linskiy IV, Minko OI, Kuzminov VN, Gmeinwieser M, Kiefer LP, et al. A qualitative assessment of insomnia in recovering alcohol-dependent patients. Neuropsychopharmacol Rep. 2023;43(4):641–6. 10.1002/npr2.12390. 37904621 10.1002/npr2.12390 PMC10739062

[31] European Union Drugs Agency (EUDA). Benzodiazepines: drug profile. Available from: https://www.euda.europa.eu/publications/drug-profiles/benzodiazepines_en. Accessed 23 December 2025.

[32] Martella M, Minutiello E, Gianino MM. Patterns of antidepressant and anxiolytic use and spending in 14 European countries (2012–2021): a comprehensive time series analysis. Health Serv Insights. 2024;17:11786329241282526. 10.1177/11786329241282526. 39386264 10.1177/11786329241282526 PMC11462615

[33] Bojanić I. Use of antidepressant and anxiolytic drugs in Scandinavian countries between 2006 and 2021: a prescription database study. Depress Anxiety. 2024;2024:5448587. 10.1155/2024/5448587. 40226718 10.1155/2024/5448587 PMC11919044

[34] Cabinet of Ministers of Ukraine. National essential medicines list. Approved by Resolution No. 333 on 25 March 2009 (as amended by Resolution No. 1081 on 13 December 2017). Available from: https://moz.gov.ua/uploads/0/3799-nacperelic_dodatok_web.pdf. Accessed 1 November 2023.

[35] DARWIN EU Coordination Centre. DARWIN EU^®^ – antipsychotic prescribing in the general population in Europe: a descriptive analysis of trends and patient characteristics. Study report P3-C1-012. Version 3.0. 25 Feb 2025. Available from: https://catalogues.ema.europa.eu/system/files/2025-03/DARWIN%20EU_Report_P3-C1-012_Antipsychotics%20general%20population_V3.pdf. Accessed 23 December 2025.

[36] Ministry of Health of Ukraine. Mental health care: what the mhGAP program gives to primary care doctors. 29 May 2023. Available from: https://moz.gov.ua/uk/ohorona-psihichnogo-zdorov%CA%BCja-scho-dae-likarjam-pervinki-programa-mhgap-. Accessed 15 January 2024.

[37] Ministry of Health of Ukraine. How to get free psychological and psychiatric assistance during the war. 11 January 2024. Available from: https://moz.gov.ua/article/news/jak-pid-chas-vijni-otrimati-bezoplatnu-psihologichnu-ta-psihiatrichnu-dopomogu-. Accessed 15 January 2024.

[38] Tkachova O, Iakovlieva L, Gerasymova O, Butko Y, Kovalenko L. Comparative analysis of the consumption of antidepressants in Ukraine, Estonia and Norway. ScienceRise Pharm Sci. 2023;3(43):23–30. 10.15587/2519-4852.2023.281833.

[39] Martsenkovskyi D, Shevlin M, Ben-Ezra M, Bondjers K, Fox R, Karatzias T, et al. Mental health in Ukraine in 2023. Eur Psychiatry. 2024;67(1):e27. 10.1192/j.eurpsy.2024.12. 38533632 10.1192/j.eurpsy.2024.12 PMC10988158

[40] Danese A, Martsenkovskyi D, Editorial. Measuring and buffering the mental health impact of the war in Ukraine in young people. J Am Acad Child Adolesc Psychiatry. 2023;62(3):294–6. 10.1016/j.jaac.2022.11.001. 36396083 10.1016/j.jaac.2022.11.001

