# Supplementary material for: The trends in the use of psychopharmacological medications in Ukraine 2010–2022
Source: BMC Psychiatry. 2026 Jan 23;26:170. doi: 10.1186/s12888-026-07835-2 (PMC12911245; doi:10.1186/s12888-026-07835-2)
Supplement: Supplementary file 1 — Supplementary Material 1: Additional file 1: Total prescriptions by year, gender, and age categorization from 2015 to 2022 [file 12888_2026_7835_MOESM1_ESM.docx]

**Additional file 7**

The most frequently dispensed antipsychotic medications, categorized by the 5th level ATC code and measured in packages from 2010 to 2022. Source: Pharmxplorer database © Research LLC, 2009-2023.

|  | 2010 | 2011 | 2012 | 2013 | 2014 | 2015 | 2016 | 2017 | 2018 | 2019 | 2020 | 2021 | 2022 |
| --- | --- | --- | --- | --- | --- | --- | --- | --- | --- | --- | --- | --- | --- |
| SULPIRIDUM | 310 340 | 307 863 | 346 115 | 364 466 | 328 939 | 284 263 | 320 271 | 319 048 | 273 002 | 415 529 | 458 495 | 526 922 | 455 421 |
| CHLORPROMAZINE | 422 386 | 377 207 | 388 498 | 385 943 | 332 608 | 290 966 | 296 077 | 311 503 | 343 748 | 293 401 | 267 940 | 240 521 | 210 903 |
| QUETIAPINUM | 5 756 | 14 023 | 28 869 | 37 673 | 47 941 | 58 622 | 93 121 | 145 040 | 195 861 | 267 534 | 343 098 | 430 904 | 447 785 |
| CLOZAPINUM | 134 060 | 143 970 | 162 731 | 192 105 | 202 606 | 184 764 | 183 869 | 198 359 | 202 539 | 202 641 | 223 510 | 232 597 | 231 578 |
| RISPERIDONUM | 52 070 | 56 087 | 71 179 | 96 192 | 89 252 | 73 836 | 85 495 | 113 049 | 127 412 | 149 662 | 175 566 | 204 636 | 207 978 |
| HALOPERIDOLUM | 151 118 | 132 211 | 134 010 | 150 830 | 137 760 | 110 820 | 110 123 | 111 424 | 92 145 | 93 129 | 97 662 | 107 116 | 91 280 |
| TRIFLUOPERAZINUM | 155 114 | 108 653 | 115 721 | 120 860 | 107 770 | 91 030 | 103 052 | 96 722 | 111 413 | 111 757 | 103 983 | 82 863 | 64 963 |
| THIORIDAZINUM | 106 126 | 98 744 | 103 139 | 82 510 | 71 585 | 39 764 | 30 353 | 33 418 | 38 437 | 38 968 | 39 585 | 42 533 | 38 763 |
| CHLORPROTHIXENE | 24 520 | 29 986 | 38 644 | 47 590 | 44 785 | 31 003 | 33 544 | 40 396 | 46 440 | 54 153 | 59 616 | 62 306 | 60 710 |
| AMISULPRIDUM | 6 029 | 11 119 | 15 917 | 19 755 | 21 518 | 20 850 | 23 765 | 31 025 | 38 513 | 45 584 | 51 409 | 59 414 | 55 210 |
