# Supplementary material for: The trends in the use of psychopharmacological medications in Ukraine 2010–2022
Source: BMC Psychiatry. 2026 Jan 23;26:170. doi: 10.1186/s12888-026-07835-2 (PMC12911245; doi:10.1186/s12888-026-07835-2)
Supplement: Supplementary file 2 — Supplementary Material 2: Additional file 2: Descriptive table for overall consumption of ATC N03-N07 nervous-system drugs from 2010 to 2022 [file 12888_2026_7835_MOESM2_ESM.docx]

**Additional file 8**

The most frequently dispensed antidepressant medications, categorized by 5th level ATC code and measured in packages from 2010 to 2022. Source: Pharmxplorer database © Research LLC, 2009-2023.

|  | 2010 | 2011 | 2012 | 2013 | 2014 | 2015 | 2016 | 2017 | 2018 | 2019 | 2020 | 2021 | 2022 |
| --- | --- | --- | --- | --- | --- | --- | --- | --- | --- | --- | --- | --- | --- |
| N06A B10 Escitalopram | 33 176 | 44 179 | 60 367 | 67 180 | 67 578 | 70 400 | 102 197 | 145 687 | 202 828 | 279 250 | 354 822 | 480 153 | 515 941 |
| N06A A09 Amitriptyline | 665 017 | 219 060 | 296 121 | 399 733 | 385 168 | 392 069 | 418 706 | 417 117 | 445 200 | 419 521 | 388 543 | 401 726 | 419 145 |
| N06A B03 Fluoxetine | 152 223 | 144 096 | 146 478 | 150 192 | 155 400 | 178 865 | 200 963 | 234 483 | 249 960 | 195 621 | 173 098 | 194 988 | 232 523 |
| N06A B06 Sertraline | 22 460 | 26 891 | 36 125 | 36 678 | 35 929 | 32 458 | 39 408 | 52 218 | 70 069 | 101 521 | 135 992 | 215 233 | 217 713 |
| N06A B05 Paroxetine | 51 281 | 68 554 | 78 283 | 91 129 | 81 536 | 69 845 | 81 565 | 99 935 | 121 115 | 150 491 | 170 198 | 204 400 | 198 287 |
| N06A X03 Mianserin | 24 009 | 41 809 | 56 248 | 64 509 | 63 195 | 66 422 | 55 263 | 112 609 | 132 851 | 44 856 | 149 447 | 202 144 | 183 779 |
| N06A X21 Duloxetine |  |  |  |  |  |  |  | 6 466 | 19 347 | 48 830 | 74 236 | 134 126 | 146 473 |
