# Supplementary material for: The trends in the use of psychopharmacological medications in Ukraine 2010–2022
Source: BMC Psychiatry. 2026 Jan 23;26:170. doi: 10.1186/s12888-026-07835-2 (PMC12911245; doi:10.1186/s12888-026-07835-2)
Supplement: Supplementary file 3 — Supplementary Material 3: Additional file 3: Descriptive table for overall consumption of a specific group of drugs from 2010 to 2022 [file 12888_2026_7835_MOESM3_ESM.docx]

**Additional file 1**

Total prescriptions by year, gender, and age categorization from 2015 to 2022. Source: Pharmxplorer database © Research LLC, 2009-2023.

| **Year** | **The total number of prescriptions** | **Sex** | **Age categorization** |
| --- | --- | --- | --- |
| 2015 | 9 249 253 | Male – 3 878 414 (41,9%)  Female – 4 194 052 (45,4%)  Unspecified – 1 176 787 (12,7%) | Males aged 1 to 18 years: 560 954  Males aged 18 to 30 years: 334 389  Males aged 30 to 40 years: 470 774  Males aged 40 to 50 years: 560 379  Males aged 50 to 70 years: 1 349 668  Over 70 years: 593 043  Females aged 1 to 18 years: 494 133  Females aged 18 to 30 years: 487 354  Females aged 30 to 40 years: 521 125  Females aged 40 to 50 years: 610 244  Females 50 to 70 years: 1 451 031  Over 70 years: 619 688  Unspecified 1 047 359  Unspecified gender 1 to 18 years: 8 865  Unspecified gender 18 to 30 years: 12 457  Unspecified gender 30 to 40 years: 13 971  Unspecified gender 40 to 50 years: 24 442  Unspecified gender 50 to 70 years: 44 407  Unspecified gender over 70 years: 25 286 |
| 2016 | 9 299 937 | Male – 4 196 694 (45,1%)  Female – 4 639 451 (49,8%)  Unspecified – 463 792 (5,1%) | Males aged 1 to 18 years: 751 933  Males aged 18 to 30 years: 403 767  Males aged 30 to 40 years: 484 914  Males aged 40 to 50 years: 563 416  Males aged 50 to 70 years: 1 306 394  Over 70 years: 677 347  Females aged 1 to 18 years: 567 512  Females aged 18 to 30 years: 520 052  Females aged 30 to 40 years: 546 606  Females aged 40 to 50 years: 644 302  Females 50 to 70 years: 1 548 861  Over 70 years: 812 118  Unspecified 463 792  Unspecified male age 8 924 |
| 2017 | 8 966 242 | Male – 4 186 269 (46,7%)  Female – 4 775 488 (53,25%)  Unspecified – 4 484 (0,05%) | Males aged 1 to 18 years: 729 516  Males aged 18 to 30 years: 386 787  Males aged 30 to 40 years: 494 359  Males aged 40 to 50 years: 571 176  Males aged 50 to 70 years: 1 289 545  Over 70 years: 714 886  Females aged 1 to 18 years: 631 905  Females aged 18 to 30 years: 564 440  Females aged 30 to 40 years: 575 732  Females aged 40 to 50 years: 639 429  Females 50 to 70 years: 1 484 853  Over 70 years: 879 129  Unspecified gender or age 4 484 |
| 2018 | 8 674 708 | Male – 4 122 876 (47,5%)  Female – 4 551 832 (52,5%) | Males aged 1 to 18 years: 760 568  Males aged 18 to 30 years: 371 914  Males aged 30 to 40 years: 486 436  Males aged 40 to 50 years: 591 870  Males aged 50 to 70 years: 1 186 264  Over 70 years: 725 824  Females aged 1 to 18 years: 624 956  Females aged 18 to 30 years: 486 120  Females aged 30 to 40 years: 505 299  Females aged 40 to 50 years: 617 471  Females 50 to 70 years: 1 424 150  Over 70 years: 893 836 |
| 2019 | 8 286 557 | Male – 3 911 380 (47,2%)  Female – 4 375 177 (52,8%) | Males aged 1 to 18 years: 702 755  Males aged 18 to 30 years: 349 283  Males aged 30 to 40 years: 466 918  Males aged 40 to 50 years: 558 494  Males aged 50 to 70 years: 1 179 283  Over 70 years: 654 647  Females aged 1 to 18 years: 519 170  Females aged 18 to 30 years: 480 761  Females aged 30 to 40 years: 525 219  Females aged 40 to 50 years: 569 489  Females 50 to 70 years: 1 460 571  Over 70 years: 819 967 |
| 2020 | 5 517 520 | Male – 2 567 429 (46,5%)  Female – 2 950 091 (53,5%) | Males aged 1 to 18 years: 482 453  Males aged 18 to 30 years: 274 434  Males aged 30 to 40 years: 353 376  Males aged 40 to 50 years: 414 410  Males aged 50 to 70 years: 681 466  Over 70 years: 361 290  Females aged 1 to 18 years: 410 192  Females aged 18 to 30 years: 344 607  Females aged 30 to 40 years: 388 575  Females aged 40 to 50 years: 408 737  Females 50 to 70 years: 958 056  Over 70 years: 439 924 |
| 2021 | 6 435 978 | Male – 2 999 250 (46,6%)  Female – 3 436 728 (53,4%) | Males aged 1 to 18 years: 569 969  Males aged 18 to 30 years: 325 455  Males aged 30 to 40 years: 430 477  Males aged 40 to 50 years: 491 360  Males aged 50 to 70 years: 802 191  Over 70 years: 379 798  Females aged 1 to 18 years: 534 131  Females aged 18 to 30 years: 496 357  Females aged 30 to 40 years: 503 746  Females aged 40 to 50 years: 528 215  Females 50 to 70 years: 931 539  Over 70 years: 442 740 |
| 2022 | 5 904 554 | Male – 2 793 475 (47,3%)  Female – 3 111 079 (52,7%) | Males aged 1 to 18 years: 439 977  Males aged 18 to 30 years: 308 142  Males aged 30 to 40 years: 400 964  Males aged 40 to 50 years: 481 548  Males aged 50 to 70 years: 725 098  Over 70 years: 437 746  Females aged 1 to 18 years: 390 387  Females aged 18 to 30 years: 410 646  Females aged 30 to 40 years: 470 692  Females aged 40 to 50 years: 452 272  Females 50 to 70 years: 898 131  Over 70 years: 488 951 |
