# Supplementary material for: The trends in the use of psychopharmacological medications in Ukraine 2010–2022
Source: BMC Psychiatry. 2026 Jan 23;26:170. doi: 10.1186/s12888-026-07835-2 (PMC12911245; doi:10.1186/s12888-026-07835-2)
Supplement: Supplementary file 4 — Supplementary Material 4: Additional file 4: The most frequently dispensed hypnotic and sedative medications (N05C), categorized by 5th level ATC code and measured in packages from 2010 to 2022 [file 12888_2026_7835_MOESM4_ESM.docx]

**Additional file 4**

The most frequently dispensed hypnotic and sedative medications, categorized by 5th level ATC code and measured in packages from 2010 to 2022. Source: Pharmxplorer database © Research LLC, 2009-2023.

|  | 2010 | 2011 | 2012 | 2013 | 2014 | 2015 | 2016 | 2017 | 2018 | 2019 | 2020 | 2021 | 2022 |
| --- | --- | --- | --- | --- | --- | --- | --- | --- | --- | --- | --- | --- | --- |
| N05C B02 Barbiturates in combination with other drugs | 25 232 757 | 23 562 175 | 23 203 018 | 21 288 393 | 17 867 753 | 14 983 504 | 14 520 742 | 14 373 187 | 13 708 591 | 12 955 939 | 12 105 029 | 11 669 700 | 10 577 982 |
| N05C M09 Valerianae radix | 12 261 694 | 13 146 235 | 12 843 922 | 12 931 250 | 13 771 851 | 12 737 219 | 12 161 920 | 12 155 552 | 10 607 998 | 9 786 978 | 8 768 285 | 7 607 925 | 7 158 584 |
| N05C M50  Other preparations, incl. combinations | 6 673 296 | 6 922 978 | 6 797 381 | 6 639 318 | 5 345 770 | 3 945 290 | 4 336 940 | 4 964 424 | 5 014 134 | 5 156 789 | 5 247 521 | 5 324 581 | 4 814 596 |
| N05C M20  Leonurus | 4 671 064 | 4 806 082 | 4 886 008 | 4 880 904 | 4 699 090 | 4 311 797 | 4 330 548 | 4 628 391 | 4 256 428 | 4 155 031 | 4 066 318 | 3 551 828 | 2 881 961 |
| N05C M23  Paeonia preparations | 546 350 | 624 539 | 640 003 | 636 677 | 642 837 | 650 445 | 629 475 | 717 822 | 666 577 | 617 456 | 650 606 | 582 321 | 517 443 |
| N05C F01 Zopiclone | 548 579 | 416 038 | 348 501 | 369 919 | 318 156 | 255 354 | 234 371 | 224 764 | 245 198 | 306 885 | 390 929 | 501 703 | 481 375 |
