# Supplementary material for: The trends in the use of psychopharmacological medications in Ukraine 2010–2022
Source: BMC Psychiatry. 2026 Jan 23;26:170. doi: 10.1186/s12888-026-07835-2 (PMC12911245; doi:10.1186/s12888-026-07835-2)
Supplement: Supplementary file 5 — Supplementary Material 5: Additional file 5: The most frequently dispensed psychostimulant medications (N06B), categorized by 5th level ATC code and measured in packages from 2010 to 2022 [file 12888_2026_7835_MOESM5_ESM.docx]

**Additional file 5**

The most frequently dispensed psychostimulant medications, categorized by the 5th level ATC code and measured in packages from 2010 to 2022. Source: Pharmxplorer database © Research LLC, 2009-2023.

|  | 2010 | 2011 | 2012 | 2013 | 2014 | 2015 | 2016 | 2017 | 2018 | 2019 | 2020 | 2021 | 2022 |
| --- | --- | --- | --- | --- | --- | --- | --- | --- | --- | --- | --- | --- | --- |
| N06B X22 Phenibut | 269 718 | 331 508 | 462 595 | 697 274 | 800 996 | 881 124 | 1 474 229 | 2 220 410 | 2 671 175 | 3 176 368 | 3 266 823 | 3 792 043 | 3 373 599 |
| N06B X06 Citicoline | 351 353 | 375 350 | 470 422 | 518 825 | 543 207 | 611 008 | 752 007 | 889 401 | 944 313 | 1 180 773 | 1 152 975 | 1 382 433 | 1 090 448 |
| N06B X18 Vinpocetine | 1 564 617 | 1 575 642 | 1 603 198 | 1 525 412 | 1 247 712 | 1 130 016 | 1 175 436 | 1 252 391 | 1 167 905 | 1 158 522 | 1 046 354 | 1 078 292 | 894 591 |
| N06B X21 Temgicoluril | 411 262 | 482 026 | 574 314 | 614 181 | 493 928 | 399 227 | 457 700 | 570 651 | 680 834 | 742 675 | 773 886 | 922 750 | 847 842 |
| N06B X53 Piracetam, combinations | 2 828 530 | 2 532 664 | 2 602 792 | 2 525 995 | 2 149 706 | 1 669 255 | 1 645 644 | 1 593 253 | 1 342 515 | 1 200 225 | 974 876 | 1 009 716 | 815 345 |
| N06B X03 Piracetam | 2 910 660 | 2 325 367 | 2 238 406 | 2 121 187 | 1 788 949 | 1 616 226 | 1 548 618 | 1 552 595 | 1 363 825 | 1 224 004 | 1 037 307 | 1 015 850 | 787 165 |
