# Supplementary material for: The trends in the use of psychopharmacological medications in Ukraine 2010–2022
Source: BMC Psychiatry. 2026 Jan 23;26:170. doi: 10.1186/s12888-026-07835-2 (PMC12911245; doi:10.1186/s12888-026-07835-2)
Supplement: Supplementary file 6 — Supplementary Material 6: Additional file 6: The most frequently dispensed anxiolytic medications (N05B), categorized by the 5th level ATC code and measured in packages from 2010 to 2022 [file 12888_2026_7835_MOESM6_ESM.docx]

**Additional file 2**

Descriptive table for overall consumption of ATC N03-N07 nervous-system drugs from 2010 to 2022. Source: Pharmxplorer database © Research LLC, 2009-2023.

| ATC code 3_Packages | 2010 | 2011 | 2012 | 2013 | 2014 | 2015 | 2016 | 2017 | 2018 | 2019 | 2020 | 2021 | 2022 |
| --- | --- | --- | --- | --- | --- | --- | --- | --- | --- | --- | --- | --- | --- |
| N05C  HYPNOTICS AND SEDATIVES | 50 200 000 | 49 700 000 | 48 900 000 | 47 000 000 | 42 900 000 | 37 100 000 | 36 500 000 | 37 400 000 | 35 000 000 | 33 500 000 | 32 100 000 | 30 800 000 | 27 600 000 |
| N06B PSYCHOSTIMULANTS, AGENTS USED FOR ADHD AND NOOTROPICS | 9 857 812 | 9 302 321 | 9 697 332 | 9 581 131 | 8 318 898 | 7 453 574 | 8 330 137 | 9 559 054 | 9 882 637 | 10 600 000 | 9 887 370 | 10 700 000 | 8 972 157 |
| N07C  ANTIVERTIGO | 3 666 771 | 3 741 529 | 4 020 774 | 4 063 100 | 3 582 243 | 3 293 672 | 3 501 386 | 3 971 309 | 3 990 887 | 4 201 703 | 3 612 636 | 4 107 073 | 3 361 941 |
| N07X  OTHER NERVOUS SYSTEM DRUGS | 2 661 783 | 3 105 491 | 4 105 490 | 4 541 115 | 4 123 469 | 3 732 672 | 3 939 891 | 4 589 578 | 4 582 374 | 4 387 244 | 3 492 778 | 3 796 209 | 2 761 264 |
| N05B  ANXIOLYTICS | 2 441 187 | 2 406 586 | 2 814 634 | 3 025 158 | 2 860 897 | 2 500 796 | 2 610 410 | 3 097 604 | 3 275 165 | 3 110 958 | 3 001 960 | 3 296 691 | 3 537 693 |
| N03A  ANTIEPILEPTICS | 2 012 805 | 1 892 006 | 2 060 058 | 2 131 197 | 1 962 071 | 1 915 180 | 2 029 229 | 2 222 965 | 2 358 413 | 2 331 981 | 2 225 446 | 2 301 607 | 2 234 279 |
| N05A  ANTIPSYCHOTICS | 1 465 771 | 1 371 660 | 1 514 928 | 1 623 487 | 1 484 958 | 1 278 341 | 1 384 137 | 1 544 621 | 1 647 383 | 1 885 796 | 2 071 374 | 2 288 759 | 2 166 124 |
| N07B  DRUGS USED IN ADDICTIVE DISORDERS | 1 446 675 | 1 341 959 | 1 402 321 | 1 431 540 | 1 121 694 | 825 350 | 898 701 | 1 148 818 | 1 332 304 | 1 709 253 | 1 662 853 | 1 805 222 | 1 305 051 |
| N06A  ANTIDEPRESSANTS | 1 199 241 | 808 342 | 1 013 873 | 1 159 726 | 1 042 583 | 973 049 | 1 093 993 | 1 305 387 | 1 506 617 | 1 570 608 | 1 814 454 | 2 293 424 | 2 331 505 |
| N06D  ANTI-DEMENTIA DRUGS | 771 428 | 792 458 | 717 158 | 753 244 | 644 632 | 533 918 | 547 691 | 649 138 | 627 850 | 641 024 | 565 363 | 601 269 | 508 009 |
| N07A PARASYMPATHOMIMETICS | 488 021 | 508 608 | 587 980 | 613 875 | 546 025 | 478 521 | 551 241 | 773 813 | 846 882 | 989 276 | 906 116 | 1 347 392 | 1 018 283 |
| N04A  ANTICHOLINERGIC AGENTS | 419 784 | 338 883 | 328 317 | 359 593 | 345 538 | 297 451 | 258 133 | 230 833 | 212 792 | 215 359 | 197 123 | 177 703 | 139 762 |
| N04B  DOPAMINERGIC AGENTS | 197 687 | 208 266 | 243 577 | 270 356 | 273 097 | 275 280 | 325 452 | 390 405 | 439 880 | 507 247 | 531 378 | 597 075 | 546 081 |
| Total ATC N | 76 800 000 | 75 500 000 | 77 400 000 | 76 500 000 | 69 200 000 | 60 700 000 | 62 000 000 | 66 900 000 | 65 700 000 | 65 600 000 | 62 000 000 | 64 200 000 | 56 400 000 |
