# Supplementary material for: The trends in the use of psychopharmacological medications in Ukraine 2010–2022
Source: BMC Psychiatry. 2026 Jan 23;26:170. doi: 10.1186/s12888-026-07835-2 (PMC12911245; doi:10.1186/s12888-026-07835-2)
Supplement: Supplementary file 7 — Supplementary Material 7: Additional file 7: The most frequently dispensed antipsychotic medications (N05A), categorized by the 5th level ATC code and measured in packages from 2010 to 2022 [file 12888_2026_7835_MOESM7_ESM.docx]

**Additional file 3**

Descriptive table for overall consumption of a specific group of drugs from 2010 to 2022. Source: Pharmxplorer database © Research LLC, 2009-2023.

| Year/packages | 2010 | 2011 | 2012 | 2013 | 2014 | 2015 | 2016 | 2017 | 2018 | 2019 | 2020 | 2021 | 2022 |
| --- | --- | --- | --- | --- | --- | --- | --- | --- | --- | --- | --- | --- | --- |
| N05C_Hypnotics and sedatives | 50 200 000 | 49 700 000 | 48 900 000 | 47 000 000 | 42 900 000 | 37 100 000 | 36 500 000 | 37 400 000 | 35 000 000 | 33 500 000 | 32 100 000 | 30 800 000 | 27 600 000 |
| Year/packages | 2010 | 2011 | 2012 | 2013 | 2014 | 2015 | 2016 | 2017 | 2018 | 2019 | 2020 | 2021 | 2022 |
| N06B_Psychostimulants, agents used for ADHD and nootropics | 9 857 812 | 9 302 321 | 9 697 332 | 9 581 131 | 8 318 898 | 7 453 574 | 8 330 137 | 9 559 054 | 9 882 637 | 10 600 000 | 9 887 370 | 10 700 000 | 8 972 157 |
| Year/packages | 2010 | 2011 | 2012 | 2013 | 2014 | 2015 | 2016 | 2017 | 2018 | 2019 | 2020 | 2021 | 2022 |
| N05B_Anxyolitics | 2 441 187 | 2 406 586 | 2 814 634 | 3 025 158 | 2 860 897 | 2 500 796 | 2 610 410 | 3 097 604 | 3 275 165 | 3 110 958 | 3 001 960 | 3 296 691 | 3 537 693 |
| Year/packages | 2010 | 2011 | 2012 | 2013 | 2014 | 2015 | 2016 | 2017 | 2018 | 2019 | 2020 | 2021 | 2022 |
| N06A_Antidepressants | 1 199 241 | 808 342 | 1 013 873 | 1 159 726 | 1 042 583 | 973 049 | 1 093 993 | 1 305 387 | 1 506 617 | 1 570 608 | 1 814 454 | 2 293 424 | 2 331 505 |
| Year/packages | 2010 | 2011 | 2012 | 2013 | 2014 | 2015 | 2016 | 2017 | 2018 | 2019 | 2020 | 2021 | 2022 |
| N05A_Antipsychotics | 1 465 771 | 1 371 660 | 1 514 928 | 1 623 487 | 1 484 958 | 1 278 341 | 1 384 137 | 1 544 621 | 1 647 383 | 1 885 796 | 2 071 374 | 2 288 759 | 2 166 124 |
