# Supplementary material for: The trends in the use of psychopharmacological medications in Ukraine 2010–2022
Source: BMC Psychiatry. 2026 Jan 23;26:170. doi: 10.1186/s12888-026-07835-2 (PMC12911245; doi:10.1186/s12888-026-07835-2)
Supplement: Supplementary file 8 — Supplementary Material 8: Additional file 8: The most frequently dispensed antidepressant medications (N06A), categorized by 5th level ATC code and measured in packages from 2010 to 2022 [file 12888_2026_7835_MOESM8_ESM.docx]

**Additional file 6**

The most frequently dispensed anxiolytic medications, categorized by 5th level ATC code and measured in packages from 2010 to 2022. Source: Pharmxplorer database © Research LLC, 2009-2023.

|  | 2010 | 2011 | 2012 | 2013 | 2014 | 2015 | 2016 | 2017 | 2018 | 2019 | 2020 | 2021 | 2022 |
| --- | --- | --- | --- | --- | --- | --- | --- | --- | --- | --- | --- | --- | --- |
| N05B A29 Gidazepamum | 1 688 378 | 1 754 343 | 2 081 031 | 2 256 597 | 2 247 013 | 2 071 219 | 2 189 189 | 2 725 108 | 3 013 199 | 2 803 051 | 2 790 071 | 2 970 949 | 3 070 524 |
| N05B B01 Hydroxyzine | 1 677 | 33 760 | 70 495 | 85 530 | 37 819 | 48 188 | 60 127 | 77 919 | 79 943 | 133 721 | 21 813 | 128 798 | 166 080 |
| N05B X04 Fabomotizole | 188 011 | 255 066 | 311 831 | 338 178 | 285 855 | 178 598 | 204 060 | 142 249 | 13 276 |  |  |  | 150 878 |
| N05B E01 Buspirone | 36 986 | 38 148 | 45 728 | 53 685 | 41 590 | 26 445 | 29 221 | 39 194 | 48 051 | 53 061 | 64 478 | 80 853 | 71 945 |
| N05B A25 Phenazepam | 252 401 | 130 727 | 126 182 | 121 528 | 101 217 | 80 730 | 58 191 | 54 206 | 54 321 | 50 149 | 50 569 | 40 499 | 29 765 |
| N05B A01 Diazepam | 270 772 | 187 264 | 171 406 | 161 993 | 141 981 | 91 832 | 62 188 | 43 494 | 46 799 | 46 915 | 45 834 | 33 508 | 24 323 |
