# Supplementary figures and images for: The trends in the use of psychopharmacological medications in Ukraine 2010–2022
Source: BMC Psychiatry. 2026 Jan 23;26:170. doi: 10.1186/s12888-026-07835-2 (PMC12911245; doi:10.1186/s12888-026-07835-2)

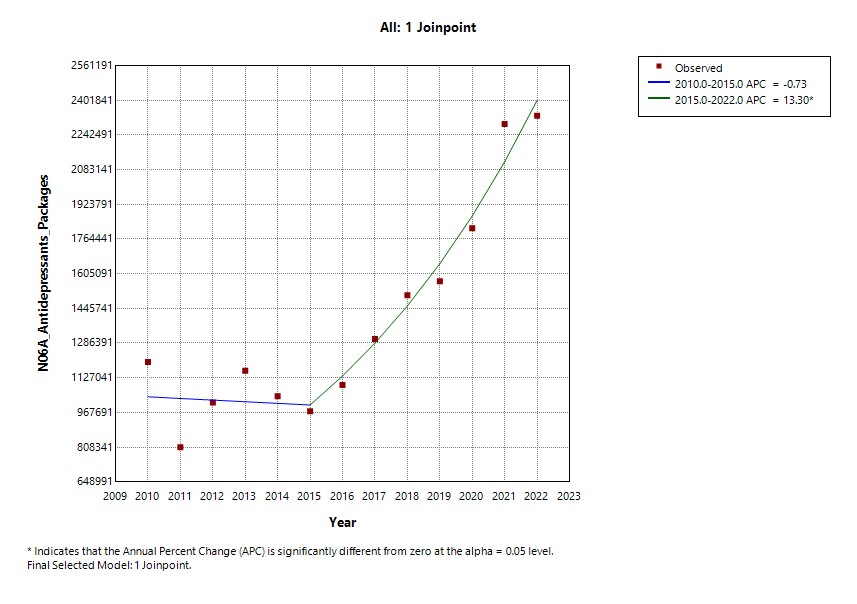

Supplement: Supplementary file 9 — Supplementary Material 9: Additional file 9: Joinpoint regression plot for hypnotics and sedatives (N05C), 2010–2022 [file 12888_2026_7835_MOESM9_ESM.jpg]

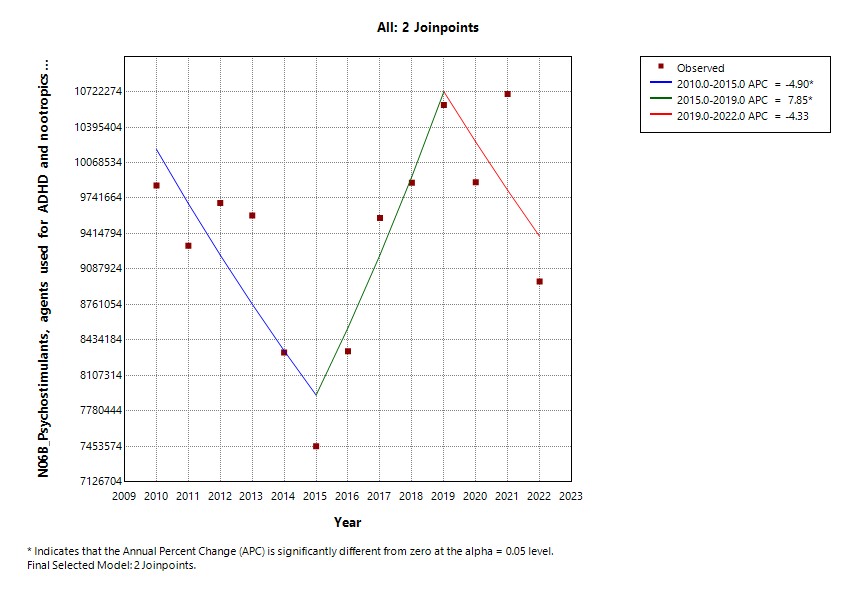

Supplement: Supplementary file 10 — Supplementary Material 10: Additional file 10: Joinpoint regression plot for psychostimulants, agents used for ADHD, and nootropics (N06B), 2010–2022 [file 12888_2026_7835_MOESM10_ESM.jpg]

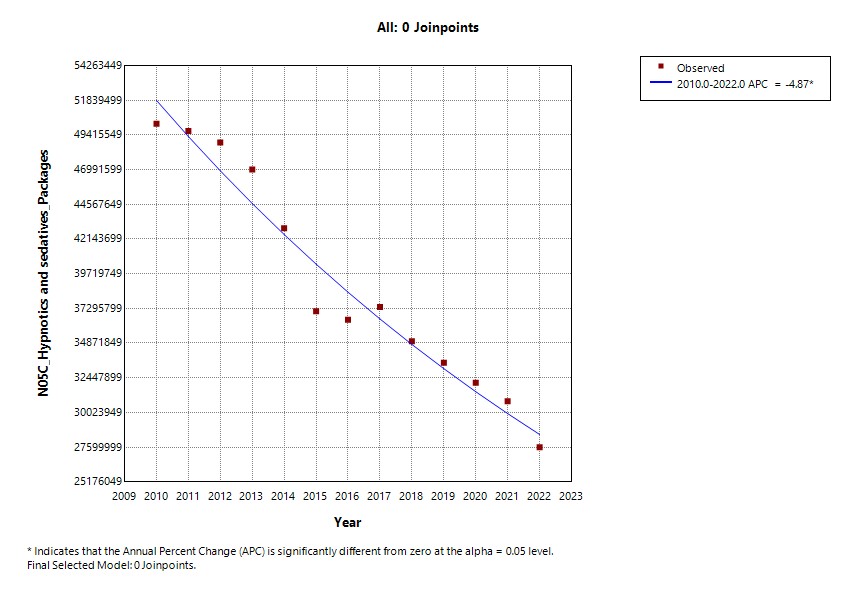

Supplement: Supplementary file 11 — Supplementary Material 11: Additional file 11: Joinpoint regression plot for anxiolytics (N05B), 2010–2022 [file 12888_2026_7835_MOESM11_ESM.jpg]

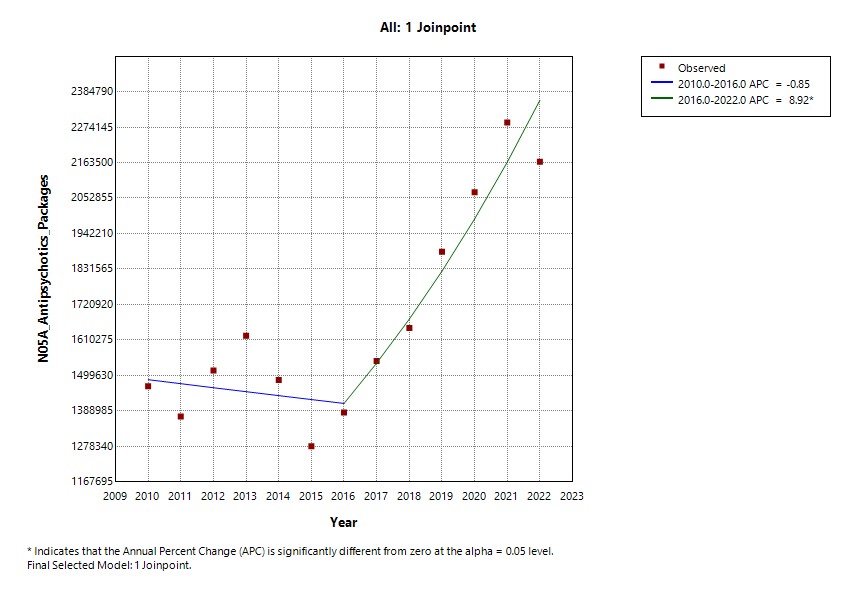

Supplement: Supplementary file 12 — Supplementary Material 12: Additional file 12: Joinpoint regression plot for antipsychotics (N05A), 2010–2022 [file 12888_2026_7835_MOESM12_ESM.jpg]

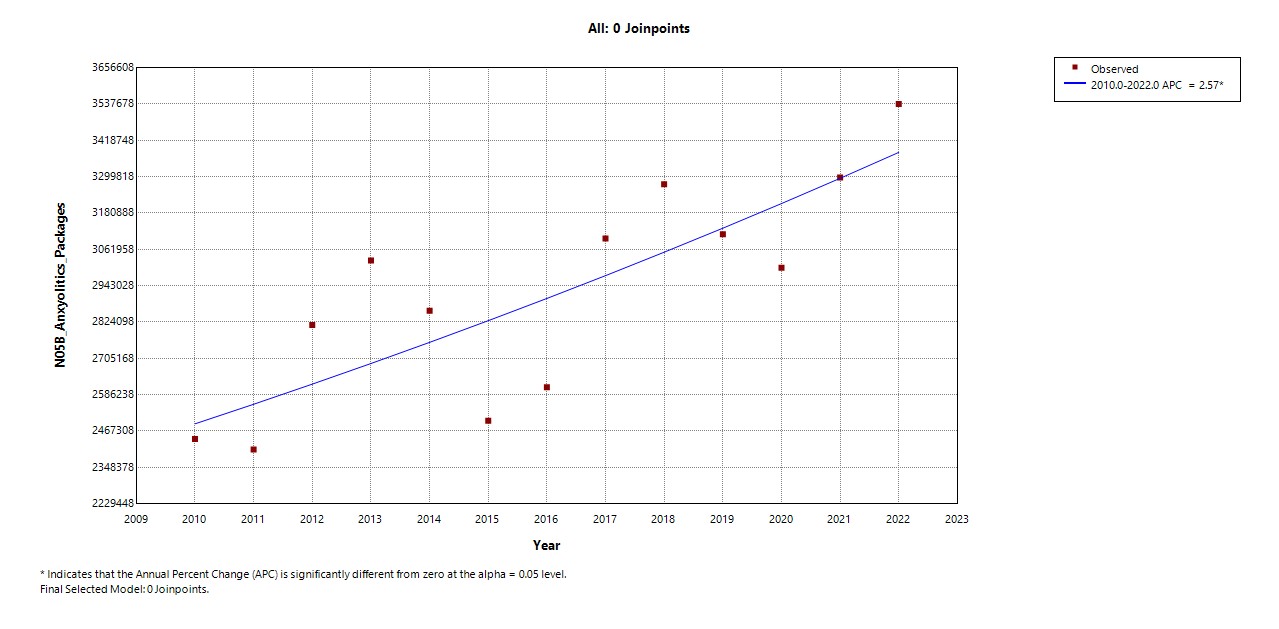

Supplement: Supplementary file 13 — Supplementary Material 13: Additional file 13: Joinpoint regression plot for antidepressants (N06A), 2010–2022 [file 12888_2026_7835_MOESM13_ESM.jpg]
